# Auxin-BR Interaction Regulates Plant Growth and Development

**DOI:** 10.3389/fpls.2017.02256

**Published:** 2018-01-18

**Authors:** Huiyu Tian, Bingsheng Lv, Tingting Ding, Mingyi Bai, Zhaojun Ding

**Affiliations:** The Key Laboratory of Plant Cell Engineering and Germplasm Innovation, Ministry of Education, College of Life Sciences, Shandong University, Jinan, China

**Keywords:** auxin, BR, crosstalk, signaling, plant

## Abstract

Plants develop a high flexibility to alter growth, development, and metabolism to adapt to the ever-changing environments. Multiple signaling pathways are involved in these processes and the molecular pathways to transduce various developmental signals are not linear but are interconnected by a complex network and even feedback mutually to achieve the final outcome. This review will focus on two important plant hormones, auxin and brassinosteroid (BR), based on the most recent progresses about these two hormone regulated plant growth and development in *Arabidopsis*, and highlight the cross-talks between these two phytohormones.

## Introduction

Unlike animals that can move to avoid the adverse surroundings, the sessile plants exhibit a highly developed adaptation to the complicated environmental conditions. To achieve this profound adaptability, communications among cells are necessary. Cell to cell communication in plants involves robust intracellular signaling processing and intricate intercellular signaling networks. Till now there are at least nine signaling substances, named plant hormones, including auxin, brassinosteroid (BR), cytokinin, gibberellins (GA), ethylene, jasmonic acid (JA), strigolactone (SL), abscisic acid (ABA), and salicylic acid (SA) discovered (Druege et al., 2016; Verma et al., 2016). The genetic and physiological studies have revealed the critical roles and functional mechanisms of these above hormones in plant growth and development (Gray, 2004). Based on the previous studies, auxin, BR, GA, SL, and cytokinin mainly function during normal plant growth and development, while ABA, ethylene, JA, and SA play important roles in plant growth response to various biotic and abiotic stresses (Pieterse et al., 2009; Santner et al., 2009; Denance et al., 2013). And also some of these hormones have dual roles, for example, ABA also plays important roles in seed development and dormancy (Seo and Koshiba, 2002). Although each hormone plays predominant roles in certain aspects, many hormones have overlapped activities and the interactions of different hormones control many developmental aspects and growth in response to endogenous developmental and exogenous cues.

Auxin and BR are two major classes of growth-promoting hormones. BR, a group of plant-specific steroid hormones which could interact with other phytohormones such as auxin, cytokinin, ethylene, GA, JA, and SA and regulate a wide range of plant growth and developmental processes including seed germination, cell elongation, vascular differentiation, stomata formation and movement, flowering and male fertility (Saini et al., 2015). Interestingly, each of these processes is also controlled by auxin, suggesting these two hormones interplay to control plant development. In this review, we will outline the signal transduction of auxin and BR based on the recent progress and review the crosstalk between auxin and BR mediated plant growth and development.

## Auxin Signaling Pathway

Auxin was first recognized as plant hormone because of its role in plant tropism to gravity or light stimuli. Later auxin was chemically identified as indole-3-acetic acid and shown to play essential roles in plethora of plant developmental and physiological processes, including embryogenesis, organogenesis, vascular differentiation, root and shoot development, tropic growth, and fruit development (Estelle, 2011).

Using genetic analysis in *Arabidopsis*, the molecular mechanism underlying the auxin signal transduction has been well investigated. TRANSPORT INHIBITOR RESPONSE1 (TIR1) was the first identified nuclear receptor of auxin (Ruegger et al., 1998; Dharmasiri et al., 2005). TIR1 encodes a nuclear protein belonging to the F-box protein as a subunit of SCF E3 ubiquitin ligase protein complex (Gray et al., 1999, 2002; Hellmann et al., 2003; Quint et al., 2005), In addition to TIR1, there are three additional F-box proteins namely AUXIN SIGNALING F BOX PROTEINs (AFBs) which show auxin-binding activity and mediate auxin signaling in *Arabidopsis* (Badescu and Napier, 2006). TIR1 receptor can interact with a group of AUX/IAA (auxin/indole-3-acetic acid) proteins (Dharmasiri et al., 2003). AUX/IAA proteins are negative regulators of auxin signaling and there are 29 members of AUX/IAA encoded in *Arabidopsis* genome. AUX/IAA proteins could interact with the class of transcriptional regulators, auxin response factors (ARF), to mediate transcriptional responses to auxin. Under high auxin level, AUX/IAA proteins interact with TIR1 as coreceptor of auxin, and can be ubiquitinated by the SCF^tir1^ complex and thus be degraded through the ubiquitin–proteasome pathway (Gray et al., 2001; Lanza et al., 2012). Upon the destruction of AUX/IAA repressors, the auxin transcriptional regulators ARFs which include 23 memberships can be released from AUX/IAA repression and thus mediate the auxin responses by activation or repression of target genes (Guilfoyle and Hagen, 2007). The different sets of F-box protein and AUX/IAA or ARFs infer the complexity during auxin signal transduction (Goh et al., 2012; Guilfoyle, 2015; Salehin et al., 2015).

The coordinated action of Aux/IAA transcriptional repressors and ARF transcription factors produces complex gene-regulatory networks which were also reported in *Physcomitrella* (Lavy et al., 2016). Recently, it was found that CULLIN1 (CUL1) subunit of the SCF interacts with TIR1 and thus regulates TIR1 substrates stability and auxin signaling (Wang et al., 2016). The interaction between TIR1 and Aux/IAA is also influenced by the spatial conformation of Aux/IAAs, controlled by a cyclophilin isomerase LRT2 in rice (Jing et al., 2015). HEAT SHOCK FACTOR 90 (HSP90) and the co-chaperone SGT1, respectively, interacts with TIR1 and thus regulates TIR1 stability, which affects the interactions between TIR1 and Aux/IAA and auxin signaling (Wang et al., 2016).

Besides the TIR1-dependent canonical auxin-signaling pathway, auxin has recently been reported to elicit a diverse range of developmental responses through a non-canonical auxin-signaling mechanism. In this non-canonical auxin sensing process, ARF3/ETTIN controls gene expression through interactions with process-specific transcription factors, which highly enriches auxin-mediated plant developmental diversity (Simonini et al., 2016, 2017).

## BR Signaling Pathway

BRASSINOSTEROID was first discovered in pollen for its ability to promote cell elongation. Later it was found that BR plays roles in a wide range of plant growth aspects and can respond to biotic and abiotic stresses. Nowadays BR signal transduction pathway was largely clarified by combinations of different methods, including molecular genetics, biochemistry, proteomics, and genomics, etc. The cell-surface kinase BRASSINOSTEROID INSENSITIVE1 (BRI1) was identified as the receptor of BR which can bind to the extracellular domain of BRI1 and activate its kinase activity and thus switch on a signal cascade to regulate transcription (Li and Chory, 1997; Wang et al., 2001; Kinoshita et al., 2005; Kim and Wang, 2010; Clouse, 2011; Hothorn et al., 2011; She et al., 2011; Oh et al., 2012). Upon perception of BR, BRI1 interacts with co-receptor BRI1-ASSOCIATED KINASE 1 (BAK1) and its homolog SOMATIC EMBRYOGENESISRECEPTOR KINASEs (SERKs) to form a more active BR receptor complex (Li et al., 2002; Nam and Li, 2002; Wang et al., 2005; Tang et al., 2008; Gou et al., 2012). Activated BRI1 phosphorylates two substrates of plasma membrane-anchored receptor-like cytoplasmic kinases: BRASSINOSTEROID-SIGNALING KINASES1 (BSK1) and CONSTITUTIVE DIFFERENTIAL GROWTH1 (CDG1) (Tang et al., 2008; Kim et al., 2011), which in turn phosphorylates a PP1-type phosphatase named BRI1-SUPPRESSOR1 (BSU1) to activate BSU1, leading to BSU1 dephosphorylation and inactivation the GSK3-like kinase BRASSINOSTEROID INSENSITIVE2 (BIN2). The kinase activity of BIN2 is also inhibited by HISTONE DEACETYLASE HDA6, which interacts and deacetylates at the K189 of BIN2. When BR levels are low, BRI1 is quiescent due to its negative regulator, BRI1 KINASE INHIBITOR 1 (BKI1) and protein phosphatase 2A (PP2A), while BIN2 phosphorylate two BR homologous transcription factors, BRASSINAZOLE RESISTANT1 (BZR1) and BZR2 (also named BES1 for BRI1-EMS-SUPPRESSOR 1) (He et al., 2002; Wang et al., 2002; Yin et al., 2002; Mora-Garcia et al., 2004; Kim et al., 2009, 2011; Kim and Wang, 2010). When BR levels are high, BIN2 is inactivated, and BZR1 and BZR2 are dephosphorylated by PP2A, and move into nucleus to alter the expression of thousands of BR response genes (He et al., 2005; Yin et al., 2005; Sun et al., 2010; Tang et al., 2011; Yu et al., 2011).

## The Synergy Between BR and Auxin Signaling

Auxin and BR signal pathways play diverse roles, however, they also showed synergistic and interdependent interactions in a wide range of developmental processes. For example, both auxin and BR signals can promote cell expansion and can interact synergistically to promote hypocotyls elongation (Nemhauser et al., 2004). The response of one of the two pathways in promoting hypocotyl elongation requires the function of the other and the interdependence between BR and auxin pathways (Nemhauser et al., 2004). Auxin increased hypocotyl length in wild-type plants but not in the BR-insensitive mutant *bri1-116*, and this auxin-insensitive phenotype of *bri1-116* was suppressed by the dominant gain-of-function mutant *bzr1-1D*, indicating BR or active BZR1 is required for auxin promotion of hypocotyl elongation. It has been found that BR signaling converges with SUPPRESSOR OF PHYTOCHROME B4-3 (SOB3) to control cell elongation and hypocotyl growth through the regulation of auxin induced *SMALL AUXIN UP RNA19* (*SAUR19*) expression (Favero et al., 2017). On the other hand, the auxin regulated transcription factor SMALL ORGAN SIZE 1 (SMOS1) has recently been found to control cell expansion through the direct interaction with SMOS2/DLT, a member of the GRAS family of transcriptional co-regulators which plays a positive role in BR signaling in rice (Kim et al., 2009; Tong et al., 2012; Hirano et al., 2017). Auxin related mutants such as *iaa3* and *arf6/arf8* were less sensitive to BR than was wild-type for hypocotyl elongation, and abolished the hypersensitivity of *bzr1-1D* to auxin, suggesting the BR and BZR1 promotion of hypocotyl elongation requires ARF6/8. The genome-wide ChIP-Seq analysis revealed that ARF6 shares a vast number of genomic targets (around 50%) with BZR1 and the light/temperature-regulated transcription factor PIF4 by CHIP-Seq analyses (Oh et al., 2014). BZR1 and PIF4 interact with ARF6 and activate shared target genes by binding to shared target genes cooperatively during hypocotyls elongation (Oh et al., 2014) and many of these overlapping target genes encode cell wall proteins involved in cell expansion.

Brassinosteroid and auxin also play important roles in the maintenance of root apical meristem (RAM) (Durbak et al., 2012). The RAM consists of a small group of rarely dividing cells known as the quiescent center (QC), surrounded by stem cells that give rise to the various toot tissue types. The maintenance of the root stem cell population is regulated by *WUSCHEL-RELATED HOMEOBOX 5* (*WOX5*) (Sarkar et al., 2007). WOX5 is restricted to the QC by auxin signaling and facilitates proper expression of the PLT genes (Aida et al., 2004; Ding and Friml, 2010). Mutations in the BR receptor gene *BRASSINOSTEROID INSENSITIVE 1* (*BRI1*) result in aberrant cell cycle progression in the RAM and cause a smaller RAMs (Gonzalez-Garcia et al., 2011; Hacham et al., 2011). Auxin is known to stimulate the biosynthesis of BR (Chung et al., 2011), but the activity of BR does not affect the expression of *PIN* genes (Hacham et al., 2011). The root tip phenotypes of BR mutants do not show the same as the auxin mutants (Gonzalez-Garcia et al., 2011), indicating that BR act on the RAM independently of auxin.

Brassinosteroid and auxin signals are also synergistically required in the radial pattern formation of vascular bundles (Ibanes et al., 2009). By the combinations of mathematical modeling and biological experiments, auxin maxima, established by asymmetric auxin polar transport, but not changes on auxin levels is important for positioning the vascular bundles. BR signal was shown to serve as a promoting signal for the number of cells in the provascular ring which are consistent with auxin maxima. Thus the establishment of periodic arrangement of vascular bundles in the shoot is under the coordinated action of these two plant hormones (Ibanes et al., 2009). Both signals are also involved in plant root development and the interaction of BR and auxin is mediated by BREVIS RADIX (BRX) during this process. BRX is important for the rate-limiting biosynthesis of BR and BR exogenous application can rescue *brx* mutant defects. Furthermore, auxin-responsive gene expression is globally impaired in *brx* mutant, and the expression of BRX is strongly induced by auxin and suppressed by BR, implying BR biosynthesis and auxin signaling are connected through a feedback loop involving BRX during root development (Mouchel et al., 2006).

Brassinosteroids and auxin also play synergistic roles during lateral root development. BRs mainly function at the lateral root primordia initiation while auxin is required for both initiation and emergence stages of lateral root formation (Casimiro et al., 2001; Bhalerao et al., 2002; Benkova et al., 2003; Bao et al., 2004). During these processes, BRs increase LRP initiation by promoting acropetal auxin transport in the root but not by affecting endogenous IAA level (Bao et al., 2004). All these reports suggest that the crosstalk between BR and auxin plays an important role in the regulation plant growth and development.

## BR Regulates Auxin Signaling

Besides the interdependency and cooperation of auxin and BR signals during plant development, BR could mediate auxin signal pathway on multiple levels. BZR1 interacts with ARF proteins to directly target multiple auxin signaling components and genes involved in auxin metabolism such as transport and signaling, including AUX/IAA, PINs, TIR1, and ARFs, etc. (Sun et al., 2010). It was found that Aux/IAA proteins are involved in BR responses and *iaa7/axr2-1* and *iaa17/axr3-3* mutants showed aberrant BR sensitivity and aberrant BR-induced gene expression in an organ-dependent manner (Nakamura et al., 2006). Exogenous brassinolide (BL) treatment could induce the expression of auxin-responsive genes such as *IAA5*, *IAA19*, *IAA17*, etc., and the expression of the above genes is down-regulated in the BR biosynthetic mutant *de-etiolated2* (*det2*), which indicates that functional BR biosynthesis is partly required for auxin-dependent gene expression (Nakamura et al., 2003; Kim et al., 2006). Additionally, BR also affects auxin flow by regulating the expression of auxin exporters such as PIN4 and PIN7 (Nakamura et al., 2004). During plant gravitropism responses, BRs could enhance the polar accumulation of the auxin exporter PIN2 in the root meristem zone and thus affect the redistribution of auxin from the root tip toward the elongation zones and result in the difference of IAA levels in both upper and lower sides of roots to induce plant gravitropism. During this process, BR activated ROP2 plays an important role in modulating the functional localization of PIN2 through the regulation of the assembly/reassembly of F-actins (Li et al., 2005). Further studies showed that decreased BL perception and/or concentration could induce CYP79B2, the gene encoding an enzyme converting tryptophan to indole-3-acetaldoxime and thus affect the distribution (Kim et al., 2007).

In addition, it was found that BR signal could regulate auxin signaling output by its negative regulator GSK3 kinase BIN2. The auxin response factor ARF2 was identified as a BIN2 interacting protein in a yeast two-hybrid screen and kinase assay showed BIN2 could phosphorylate ARF2. The phosphorylation of ARF2 results in the loss of its DNA binding ability and repression activity of the target genes (Vert et al., 2008). *ARF2* is a BZR1 target genes and its expression is reduced by BR treatment (Sun et al., 2010). Additionally, BIN2 can phosphorylate ARF7 and ARF19 to suppress their interaction with AUX/IAAs and thereby enhance the transcriptional activity on their target genes LATERAL ORGAN BOUNDARIES-DOMAIN16 (LBD16) and LBD29 to regulate lateral root organogenesis (Cho et al., 2014). However, BR plays a minor role during this process and BIN2 is under the control of the TRACHEARY ELEMENT DIFFERENTIATION INHIBITORY FACTOR (TDIF)–TDIF RECEPTOR (TDR) module (Cho et al., 2014). Together, BR can regulate auxin reponses through influencing different auxin signaling components.

## Auxin Regulates BR Signaling

On the other hand, auxin can also regulate BR signal pathway in certain aspects. The expression of *DWARF4*, a crucial hydroxylase for BR biosynthesis to control endogenous BR level, is auxin dependent. Auxin treatment could noticeably stimulate the expression of *DWARF4* and auxin could inhibit the binding of BZR1 to the promoter of DWARF4. The induction of *DWARF4* by auxin requires auxin signaling pathway but not BR signaling pathway (Chung et al., 2011; Yoshimitsu et al., 2011). CPD catalyzing C-3 oxidation of BR was activated by BRX, a putative transcription factor acting downstream of auxin signaling (Mouchel et al., 2006). Further study in rice indicates that exogenous auxin can enhance the transcription expression levels of BR receptor gene *OsBRI1*, suggesting that auxin enhances BR signaling through the regulation of BR receptors (Sakamoto et al., 2013). Furthermore, the promoter of *OsBRI1* possesses an upstream auxin-response element (AuxRE) motif which is targeted by ARF transcription factors. Moreover, mutant studies indicate that upon mutation of AuxRE, the induction of expression of *OsBRI1* by auxin is abolished and also the expression of *OsBRI1* is down regulated in *arf* mutant (Sakamoto et al., 2013). It has been reported that OsARF19 binds to the promoter of OsBRI1 and positively regulates its expression which then activates the BR signaling (Zhang et al., 2015). BES1 can bind to the promoter of SMALL AUXIN-UP RNA 15 (SAUR15) and mediate BR early response gene in *Arabidopsis*, and this binding could be enhanced by auxin treatment (Walcher and Nemhauser, 2012). Taken together, auxin can also affect BR responses and BR regulated plant growth and development.

## Concluding Remarks and Future Perspective

**FIGURE 1:**
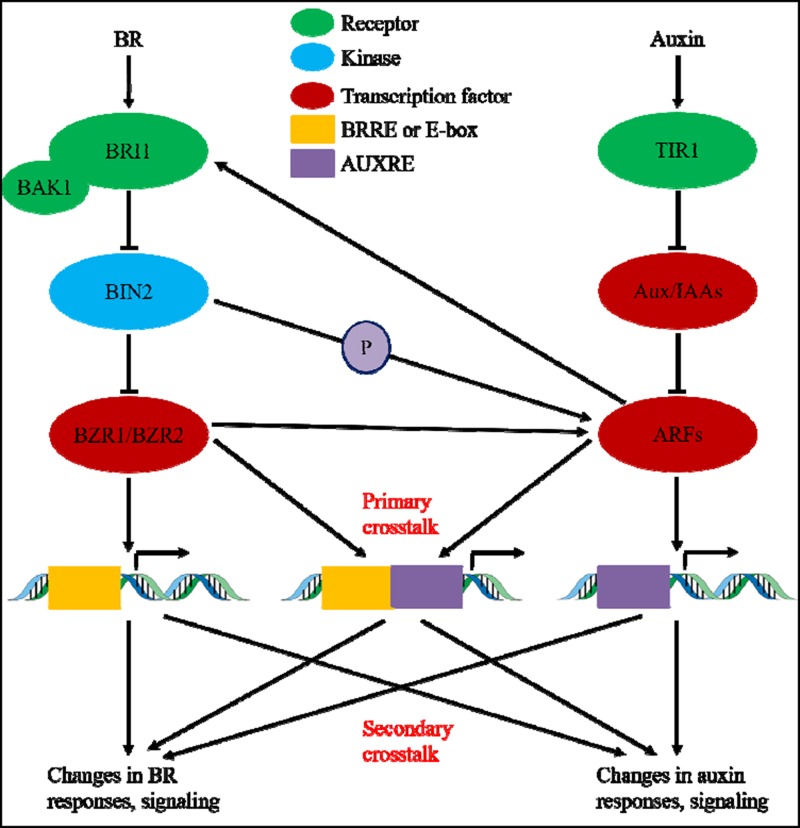
Model of auxin-brassinosteroid (BR) crosstalk. In *Arabidopsis*, the perceptions of BR and auxin signal are recognized by BRI1 and TIR1 receptors, respectively. BR binds to the extracellular domain of BRI1 and promote it interacts with co-receptor BAK1 to form a more active BR receptor complex, which in turn lead to the dephosphorylation and inactivation of BIN2. The inactivation of BIN2 lead to the dephosphorylation of two BR homologous transcription factors BZR1 and BZR2, which move into nucleus to activate transcription of genes containing BRRE or E-box in their promoter region. BIN2 also can phosphorylate ARF7 and ARF19 to suppress their interaction with AUX/IAAs and thereby enhance the transcriptional activity on their target genes. TIR1 receipt the auxin signaling and interact with AUX/IAA proteins as co-receptor of auxin. The AUX/IAA then is degraded through ubiquitin–proteasome pathway, and the auxin transcriptional regulators auxin response factors (ARFs) are released from AUX/IAA repression and activate transcription of genes with auxin responsive elements (AUXRE) in their regulatory region. Some ARFs can also binds to the promoter of BRI1 and positively regulates its expression which then activates the BR signaling. Primary crosstalk occurs by activation of genes that contain both BRRE/E-box and AUXRE in their promoter region, allowing both signaling pathways to directly regulate transcription. Secondary crosstalk occurs through expression of genes that are either auxin or BR responsive, but the activities of which control expression of genes that regulate the response and signaling of other hormones.

During the past nearly four decades, studies on auxin-BR pathway interactions have attracted more and more researchers’ interest. The appliance of physiological, molecular, genetic, and biochemical tools have greatly deepened our understanding of this issue. Based on the previous studies, BR and auxin are involved synergistically in multiple plant developmental processes including: hypocotyl elongation, vascular bundles development, root development and tropisms, etc. The interdependency and cooperation of auxin and BR are complicated and involve numerous processes on the molecular level, by sharing the same target genes, regulating each other mutually on multiple levels (**Figure 1**).

Phosphorylation regulation plays a crucial role in BR signaling pathway, especially during the perception process, BR is perceived through BRI1 kinase receptor and BAK1 kinase co-receptors, and eventually controls BR regulated gene expression through influencing downstream transcription factors such as BES1/BZR1 activities (He et al., 2005; Yin et al., 2005; Sun et al., 2010; Tang et al., 2011; Yu et al., 2011). However, ubiquitination regulation seems essential for auxin signaling. Once auxin binds to TIR1 receptor, which acts as an ubiquitin E3-ligase, the activated TIR1 E3-ligase ubiquitinates AUX/IAA proteins, leads to the degradation of these repressors and de-represses ARF transcription factors, and eventually causes auxin regulated gene expression pattern changes and growth responses (Gray et al., 1999, 2002; Hellmann et al., 2003; Quint et al., 2005). Since it has been found that BIN2 kinase, which is well known functioning in BR signaling, could phosphorylate and enhance the activities of ARFs such as ARF2 and ARF7 (Vert et al., 2008; Cho et al., 2014), it will be interesting to test if kinases such as BIN2, which are involved in BR signaling, could also interact with other auxin signaling components such as TIR1 receptor or AUX/IAA repressors, and influence TIR1 E3-ligase activity or AUX/IAA protein stabilities. On the other hand, the role of ubiquitination in BR signaling also needs to be addressed, especially if TIR1 E3-ligase could directly interact with BR signaling components and regulate their protein stabilities.

In addition, using auxin response DR5 and other auxin reporters, it has been observed that auxin regulates plant growth and development in a tissue or cellular dependent manner. The diverse transcriptional outputs depending on the cellular and environmental context (Clark et al., 2014; Etchells et al., 2016; Lavy et al., 2016). Though the spatiotemporal BR signaling has been shown to control root growth through the antagonistic action with auxin (Chaiwanon and Wang, 2015), it is still unknown if the tissue or cellular BR signaling, which could be visualized by pBZR1:BZR1-YFP, is also important to control other processes besides root development. Furthermore, generation of a detailed tissue or cellar map of auxin and BR distributions is currently possible using fluorescence-activated cell sorting or laser microdissection in combination with high-resolution gene expression analysis. This will eventually leads to address if the auxin crosstalks with BR in a tissue or cellular manner.

## Author Contributions

All authors were involved into the writing of this review manuscript. For more information on what constitutes authorship, please refer to our author guidelines.

## Conflict of Interest Statement

The authors declare that the research was conducted in the absence of any commercial or financial relationships that could be construed as a potential conflict of interest.
